# An unexpected discovery of a novel potentially pathogenic APP gene variant: a case report of slowly progressive Alzheimer’s disease with prominent cerebral amyloid angiopathy

**DOI:** 10.3389/fnins.2025.1703718

**Published:** 2026-01-02

**Authors:** Matyas Sykora, Marie Harmackova, Eva Parobkova, Robert Rusina, Radoslav Matěj

**Affiliations:** 1 Department of Neurology, Thomayer University Hospital, Prague, Czechia; 2 BrainBank, Third Faculty of Medicine, Charles University and Thomayer University Hospital, Prague, Czechia; 3 Department of Pathology and Molecular Medicine, Third Faculty of Medicine, Charles University and Thomayer University Hospital, Prague, Czechia; 4 Department of Neurology, Faculty of Medicine, Charles University and University Hospital Hradec Kralove, Prague, Czechia; 5 Department of Pathology, First Faculty of Medicine, Charles University and General University Hospital, Prague, Czechia

**Keywords:** Alzheimer’s disease, amyloid precursor protein, cerebral amyloid angiopathy, variant, neurodegeneration

## Abstract

Amyloid precursor protein (APP) plays an essential role in brain function and development. Variants in the APP gene are associated with both familial Alzheimer’s disease and cerebral amyloid angiopathy. We report a case of early onset, slowly progressive mixed dementia with a newly identified APP variant. The patient developed mild cognitive impairment at age 51, followed by neuropsychiatric symptoms, seizures, and progressive white matter changes. Despite a fluctuating clinical course, significant deterioration occurred later, culminating in death at age 77. Genetic testing revealed an APP c.2086G > A (p.Gly696Ser) variant, currently classified as a variant of uncertain significance (VUS). *Postmortem* examination showed definite AD neuropathologic changes, with fully blown amyloid pathology including amyloid deposits in plaques as well as in severe generalized cerebral angiopathy with concomitant advanced FTLD-tau pathology. *In silico* analysis of the variant’s impact was performed, and the inconclusive results are discussed later.

## 1 Introduction

Amyloid precursor protein (APP) is a widely expressed type I transmembrane protein. While accumulation of amyloid-β (Aβ) in the form of extracellular plaque is considered pathological, APP itself has diverse and essential biological functions, including roles in cell signaling, intracellular transport, neuronal development, synaptogenic, and neuroprotective functions (Tang et al., 2016; Zheng and Koo, 2011). Variants of APP also play key roles in the pathogenesis of both sporadic and familial forms of AD.^1^

APP undergoes proteolytic processing through two distinct pathways, initiated by either α- or β-secretase, each leading to different carboxy-terminal fragments (CTFs). In the non-amyloidogenic pathway, APP is initially cleaved by α-secretase, resulting in the production of a soluble ectodomain and a membrane-bound CTF (α-CTF). Subsequent cleavage of α-CTF by γ-secretase generates the P3 peptide and the APP intracellular domain (AICD) (Cha et al., 2022; Chow et al., 2010; Zheng and Koo, 2011). In the amyloidogenic (β-secretase) pathway, APP is first cleaved by β-secretase at the precise N-terminus of the Aβ sequence, generating a membrane-bound fragment known as C99. This fragment is subsequently processed by γ-secretase, resulting in the release of the mature amyloid-β (Aβ) peptide, which ranges from 38 to 43 amino acids in length. β-Secretase processing leads to the production of potentially pathogenic Aβ species, including Aβ42, which is associated with AD (Vassar et al., 2009).

Cerebral amyloid angiopathy (CAA) is a less common primary cause of dementia, characterized by pathological Aβ deposition in the walls of cerebral blood vessels. In advanced stages, CAA can lead to intracerebral hemorrhage or cerebral ischemia (Greenberg et al., 2020). While commonly associated with aging, hereditary forms linked to APP variants have also been reported. Genetic risk factors include variants in the APP gene and the presence of the apolipoprotein E (ApoE) ε4 allele (Cortini et al., 2018; Greenberg et al., 2020).

Despite progress in understanding the neuropathological mechanisms underlying CAA, disease-specific diagnostic tools and effective disease-modifying treatments remain limited, and further research is needed.

## 2 Case description

### 2.1 Introduction: clinical background

A 56-year-old woman without a family history of neuropsychiatric disorders or dementia began experiencing symptoms of memory impairment and depressive symptoms in 2003. She underwent follow-up assessments until her death in 2024, at the age of 77. The patient completed secondary education, and professionally, she worked as a tailor for most of her life. She lived in a functional family environment. According to available information, she had been retired on an old-age pension since 2012 (at the age of 65). According to available psychological assessments, the patient’s premorbid intellectual functioning was within the above-average cognitive range.

**Figure 1:**
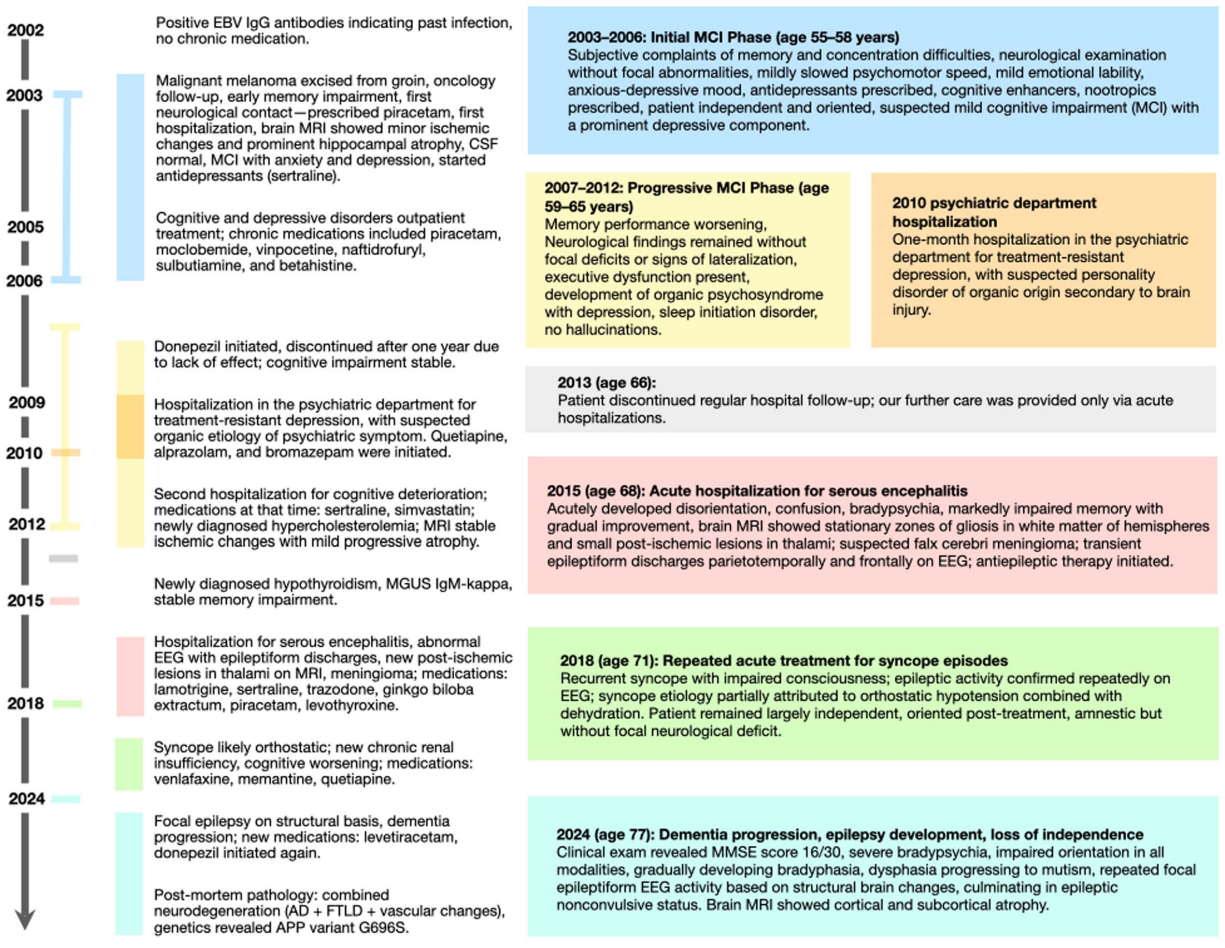
Important clinical milestones and progression details are outlined in the comprehensive timeline.

The patient’s medical history is notable for Epstein–Barr virus (EBV) infection, recurrent depressive episodes, hypothyroidism, dyslipidemia, and chronic kidney disease. Additional clinical milestones and progression details are outlined in the comprehensive timeline (Figure 1).

Initial neuropsychological testing revealed deficits in attentional persistence, reduced nonverbal memory capacity, diminished visual memory, increased fatigability, and a tendency toward perseveration. During the first 10 years, the patient was regularly monitored; thereafter, clinical contact was limited to acute evaluations or hospitalizations associated with sudden clinical deterioration. Throughout the entire period, the patient was concurrently followed by an outpatient psychiatrist for depressive syndrome. The Geriatric Depression Scale (GDS) consistently yielded scores ≥10, indicating clinically significant depressive syndrome. In later years, psychiatric follow-up was expanded to include monitoring for cognitive decline.

Cognitive symptoms were first reported by the patient in 2003 (at the age of 56), with objective findings indicating deficits in visual and nonverbal memory, moderate depressive symptoms, and progressive psychomotor slowing. Over time, further deterioration was observed in declarative memory and executive functioning, with a contributory affective component.

Beginning in 2010 (at the age of 63), the patient exhibited marked progression of executive dysfunction, with decreased attention, reduced learning capacity, bradypsychia, bradyphasia, and ultimately progressing to aphasia in the terminal phase. In 2015 (at the age of 68), the patient experienced her first seizure with epileptiform activity detected on subsequent EEGs. From 2018 onward (71 years old), recurrent orthostatic collapses were documented, and in 2024 (77 years old), the patient entered nonconvulsive status epilepticus. The patient died in 2024 at the age of 77 years from intercurrent infection.

### 2.2 Examination results

#### 2.2.1 Physical examination

Throughout the follow-up, the patient exhibited no gait disturbances, Parkinsonism, tremor, meningeal signs, or focal cerebellar or pyramidal neurological deficits. The clinical picture was primarily characterized by progressive cognitive decline accompanied by affective disturbance with organic depression.

#### 2.2.2 Laboratory

Initial tests were within normal limits. In 2002 (at the age of 55), EBV IgG positivity confirmed prior infection. Cerebrospinal fluid (CSF) analysis in 2003 showed no oligoclonal bands, no Borrelia-specific antibodies, and no evidence of central nervous system inflammation. Repeated immunological screening was negative.

In 2009 (at the age of 62), hypercholesterolemia was confirmed and treated with statins. Subsequently, hypothyroidism developed. Genetic testing revealed an ApoE genotype of E3/E4, associated with a modestly elevated risk for AD.

In 2012 (at the age of 65), monoclonal gammopathy of undetermined significance (MGUS) of the IgM-kappa subtype was identified. Immunoglobulin levels remained stable, and no Bence-Jones proteinuria was observed. During a 2015 hospitalization for aseptic encephalitis, a CSF analysis found mild pleocytosis; however, cultures and viral PCR were negative, suggesting postinfectious or autoimmune encephalitis.

#### 2.2.3 Imaging

**Figure 2:**
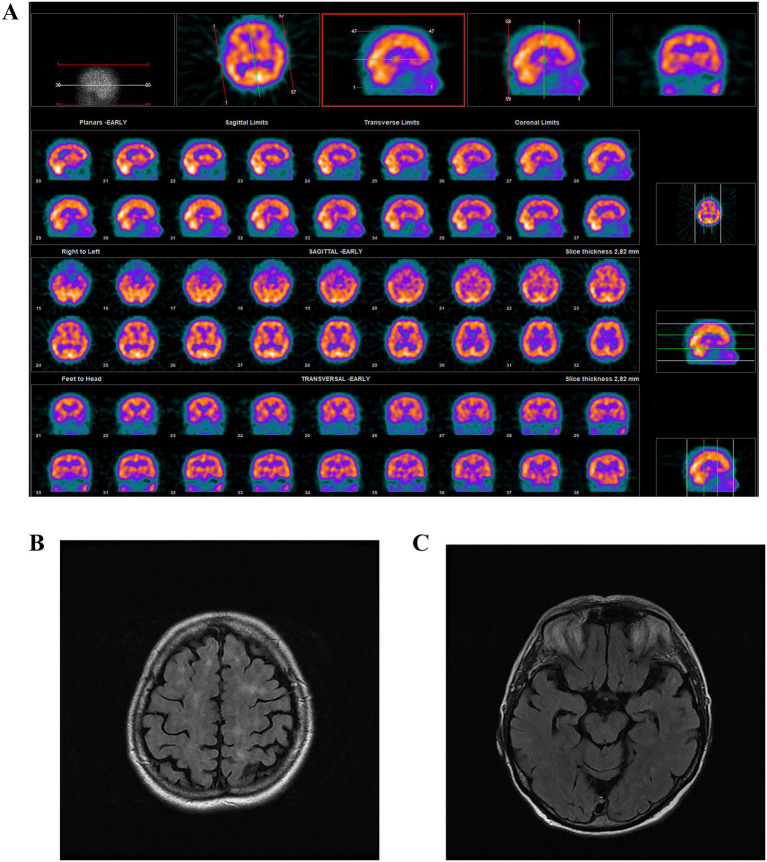
SPECT imaging **(A)** showed diffusely reduced cortical activity, with borderline findings suggestive of global cortical hypometabolism. MRI, from 2015 **(B,C)**, showed significant cortical and subcortical atrophy, predominantly in the temporal lobes and hippocampi, with focal chronic ischemic white matter lesions resulting from microangiopathy. Periventricular hyperintensities on T2/FLAIR MRI, categorized according to vascular involvement.

Serial brain imaging demonstrated progressive structural changes consistent with a neurodegenerative process. An early brain MRI from 2004 (at the age of 57) demonstrated focal signal abnormalities in the hippocampal regions, hippocampal atrophy, elongation of intracranial arteries, and supratentorial gliosis. From 2009 onward, repeated MRIs revealed progressive frontoparietal corticosubcortical atrophy, mild ventricular enlargement, and widespread small periventricular white matter microvascular changes with suspected hemosiderin deposits, supporting a diagnosis of mixed dementia. SPECT imaging (Figure 2A) showed diffusely reduced cortical activity, with borderline findings suggestive of global cortical hypometabolism. The last available MRI, from 2015 (at the age of 68) (Figures 2B,C), showed significant cortical and subcortical atrophy, predominantly in the temporal lobes and hippocampi, with focal chronic ischemic white matter lesions resulting from microangiopathy. Periventricular hyperintensities in T2/FLAIR sequences had progressed from those seen in previous scans, indicating ongoing vascular involvement.

#### 2.2.4 Therapy

At the time of the first evaluation in 2003, the patient was not receiving any chronic pharmacological treatment. Due to significant depressive symptoms, sertraline and piracetam were initiated at this time. Over subsequent years, both antidepressant and cognitive therapy regimens underwent frequent modifications. In response to extensive vascular white matter lesions, vasoactive agents were also incorporated into the therapeutic strategy. By 2005, the patient was undergoing chronic pharmacological management with piracetam, moclobemide, vinpocetine, naftidrofuryl, sulbutiamine, and betahistine. Over time, several of these agents were discontinued based on clinical judgment, primarily due to limited therapeutic efficacy or an unfavorable benefit-to-risk assessment. Donepezil was trialed in 2006–2007 without significant clinical benefit and subsequently discontinued. In the ensuing years, quetiapine, alprazolam, and bromazepam were introduced based on psychiatric evaluation and recommendation. By 2015, pharmacotherapy had been significantly expanded to include lamotrigine as an antiseizure medication, as well as EGb 761 (standardized *Ginkgo biloba* extract), trazodone, piracetam, and levothyroxine. In 2018, venlafaxine and memantine were introduced. In 2024, levetiracetam was started for structural focal epilepsy, and donepezil was reintroduced. The patient repeatedly declined psychotherapeutic interventions.

#### 2.2.5 Treatment outcomes and challenges

The overall therapeutic response remained suboptimal, prompting recurrent adjustments to the pharmacological regimen due to insufficient clinical efficacy. The patient experienced only partial or transient symptomatic improvement in cognition and mood. Pharmacotherapy remained primarily symptomatic in nature, with a steadily progressive clinical course. Treatment adherence was suboptimal, possibly exacerbated by frequent medication changes across multiple providers. Overall, medication tolerability was mostly good, although some drugs were discontinued due to inefficacy (e.g., donepezil). No severe adverse drug reactions were documented, though recurrent episodes of syncope may have been partly attributable to quetiapine-associated side effects.

#### 2.2.6 Follow-up testing results

##### 2.2.6.1 Postmortem

**Figure 3:**
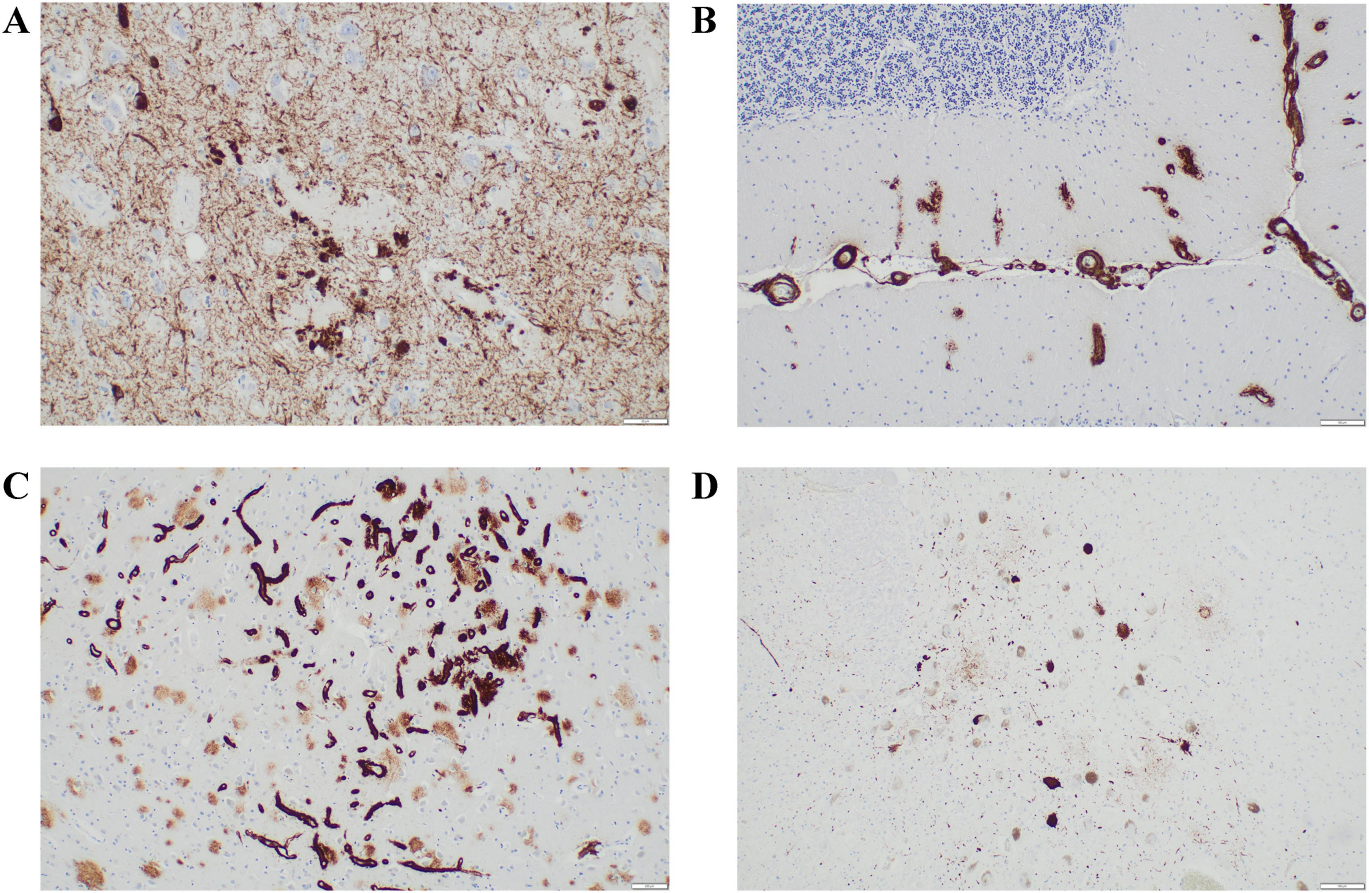
A comprehensive neuropathological and immunohistochemical examination, revealed a combination of neurodegenerative entities along with remarkable vascular changes. According to the revised “ABC” classification by the NIA, ADNPC were classified as “intermediate” due to limited tau pathology (Braak stage IV); **(A)** despite extensive amyloid deposition (Thal phase 5, CERAD score C), including amyloid-β peptide deposits in the cerebellum **(B)**, as well as severe generalized cerebral CAA. CAA was prominent across multiple vessel types, characterized by the formation of amyloid aggregates **(C)**. Additionally, widespread deposits of hyperphosphorylated tau protein of multiple isoforms were identified across distinct brain regions **(D)**. These findings supported a diagnosis of combined FTLD with tau-positive inclusions, consistent with FTLD-tau/tauopathy.

The autopsy, including a comprehensive neuropathological and immunohistochemical examination, revealed a combination of neurodegenerative entities along with remarkable vascular changes. According to the revised “ABC” classification by the National Institute on Aging (NIA), AD-associated neuropathological changes (ADNPC) were classified as “intermediate” due to limited tau pathology (Braak stage IV; Figure 3A) despite extensive amyloid deposition (Thal phase 5, CERAD score C), including amyloid-β peptide deposits in the cerebellum (Figure 3B), as well as severe generalized CAA.

CAA was prominent across multiple vessel types, characterized by the formation of amyloid aggregates (Figure 3C). Additionally, widespread deposits of hyperphosphorylated tau protein of multiple isoforms were identified across distinct brain regions (Figure 3D). These findings supported a diagnosis of combined frontotemporal lobar degeneration (FTLD) with tau-positive inclusions, consistent with FTLD-tau/tauopathy.

**Figure 4:**
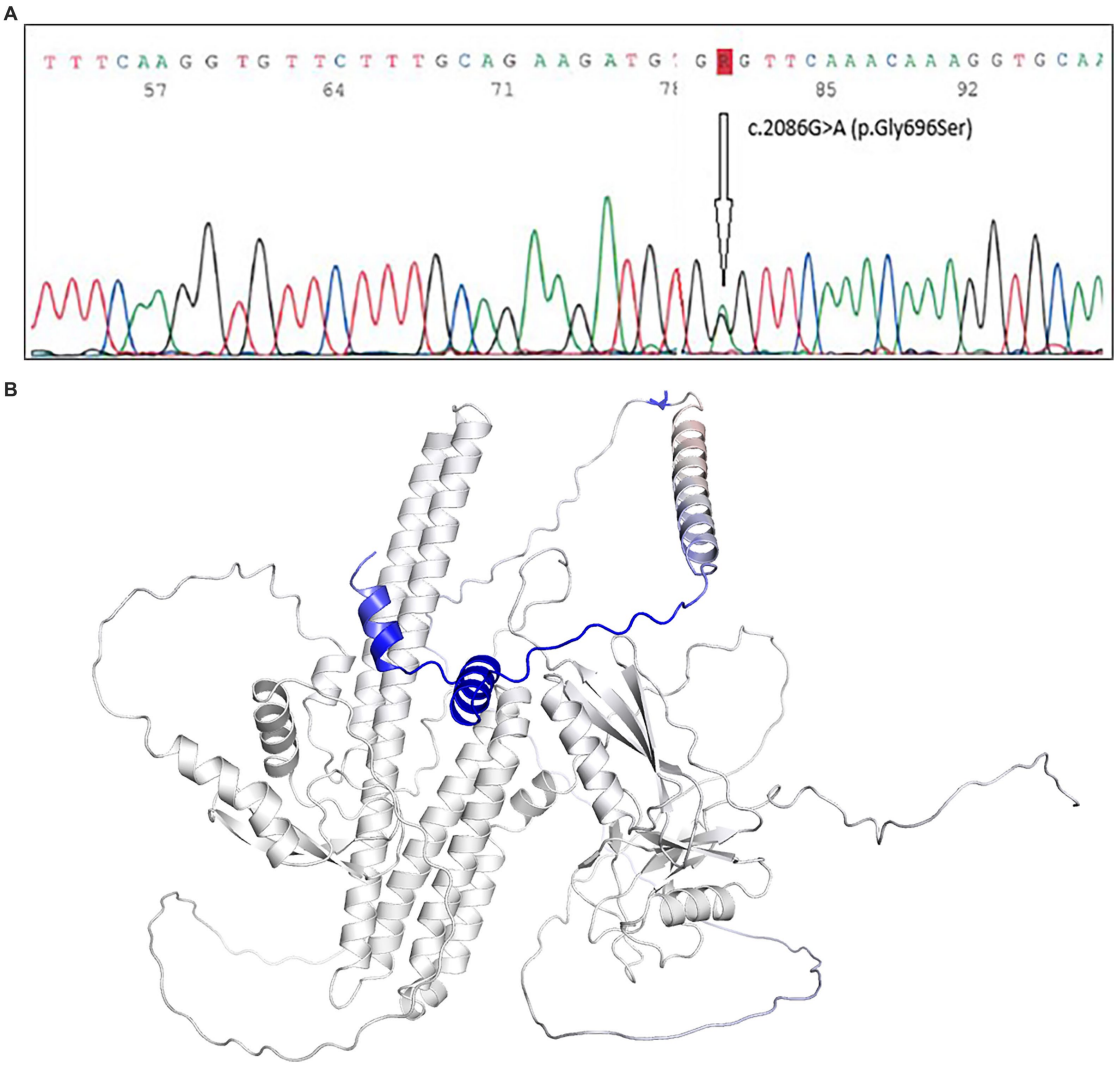
**(A)** Genetic testing revealed an APP c.2086G > A (p.Gly696Ser) variant, classified in the ClinVar database as a variant of uncertain significance (VUS). **(B)** The DynaMut (Rodrigues et al., 2018) prediction outcome established the variant impact as stabilizing – with a decrease in molecule flexibility (*Δ* Vibrational Entropy Energy Between wild-Type and mutant ΔΔS_Vib_ ENCoM: −0.287 kcal.mol^−1^.K^−1^).

Genetic testing revealed an APP c.2086G > A (p.Gly696Ser) variant (Figure 4A), classified in the ClinVar database as a variant of uncertain significance (VUS).

## 3 Discussion

### 3.1 Differential diagnostic considerations

The patient’s clinical course was one of progressive cognitive decline since 2003. MRI revealed hippocampal atrophy and leukoaraiosis; the presence of the ApoE E3/E4 risk genotype and vascular risk factors supported a diagnosis of mixed dementia, that is, AD with a prominent vascular component. A postencephalitic etiology related to a prior suspected EBV infection was initially considered in the differential but remained doubtful throughout the clinical course. A definitive diagnosis of mixed dementia was confirmed *postmortem.*

In the early years, the patient was diagnosed with a mood disorder accompanied by organic depression, correlating with white matter abnormalities observed on MRI. With disease progression, the diagnosis evolved towards mixed dementia with neuropsychiatric manifestations.

The patient’s prognosis was therefore considered poor due to several converging factors: the early onset and steadily progressive nature of cognitive decline, the presence of structural brain atrophy and vascular pathology on neuroimaging, seizures, the presence of the ApoE E3/E4 genotype, and significant vascular comorbidities.

### 3.2 Pathogenicity of novel variant

A previously reported variant in the APP gene – c.2086G > A (p.Gly696Ser) – which had been identified in another patient by commercial laboratory by 2023 an uploaded as VUS, was confirmed in this patient with postmortem-verified early-onset AD. The clinical picture was accompanied by vascular dementia with excessive CAA, and despite the high-risk features, the disease course was unusually slow. According to the ACMG/AMP classification criteria (Richards et al., 2015), the variant meets the following supporting evidence criteria (Table 1).

**Table 1:** Supporting evidence criteria according to the ACMG/AMP.

PM2 (moderate)	The variant is absent or extremely rare in population databases (e.g., gnomAD).
PM1 (moderate)	The mutation lies within a well-characterized functional domain, which is a known hotspot for pathogenic variants.
PP4 (supporting)	The clinical phenotype is highly specific and consistent with the effects of known pathogenic *APP* mutations.
PP3 (supporting)	Multiple line computational evidence supports a deleterious effect on the gene or gene product.

According to the ACMG/AMP classification criteria, the variant meets the following likely pathogenic (LP) supporting evidence criteria. Adopted from: Standards and guidelines for the interpretation of sequence variants: a joint consensus recommendation of the American College of Medical Genetics and Genomics and the Association for Molecular Pathology (Richards et al., 2015); PM, pathogenic moderate; PP, pathogenic supporting.

Currently, based on the available evidence, the functional significance of the variant has not been experimentally verified and is therefore classified as a variant of uncertain significance (VUS). Additionally, the specific pathophysiological mechanism and clinical impact of the variant remain unclear. Notably, the early onset of cognitive decline aligns with the known pathogenic potential of APP variants; however, the slow progression over two decades is certainly atypical in the context of intermediate ACNCP and CAA. Intermediate ADNPC, marked by extensive amyloid deposition in all investigated brain areas, as well as in vascular walls, reflects a strikingly generalized CAA pattern, which may have been causally linked to the novel pathological variant in the APP gene. Along with additional concomitant deposits of hyperphosphorylated tau, the variant likely impacts the amyloidogenic pathway of the APP gene; this observation may represent indirect proof of the pathogenicity of the variant. In our case, a marked deposition of amyloid-β peptide and extensive CAA in with an APP (Gly696Ser) variant represents a phenotype–genotype correlation, which, according to ACMG/AMP criteria, could be supporting (PP4) evidence of pathogenicity. The variant is located directly in the Aβ sequence, close to the transmembrane domain and a known hotspot of pathogenic variants, approximately 12–15 bp from the γ-secretase cleavage site of AICD.

Moreover, localization of this variant is in a conserved region of APP where other substitutions (e.g., E693G, A692G) have previously been reported as pathogenic mutations associated with familial AD and CAA. This supports the functional importance of the Gly696 residue and suggests that a Ser substitution at this position may also have a substantial functional impact (ACMG criteria PM1/PM5).

Due to the absence of clinical data, we estimated the stability of the mutated protein *in silico*. Based on the data extracted from (Douglas et al., 2014) (∆∆G = −0.748 kcal/mol), the negative Gibbs free energy value indicates that the substitution of Gly with Ser leads to a slightly unstable APP molecule. We used AlphaMissense (Cheng et al., 2023), a reliable tool for predicting the effects of missense mutations across the proteome. The tool classified the variant as likely benign, with a score of 0.066. Nevertheless, both results rely on predictive algorithms and represent probabilistic estimates. Notably, the performance of AlphaMissense may be limited when assessing potential gain-of-function (GoF) mutations (Johnson et al., 2023). Finally, the DynaMut (Rodrigues et al., 2018) prediction outcome established the variant impact as stabilizing – with a decrease in molecule flexibility (Δ Vibrational Entropy Energy Between wild-Type and mutant ΔΔS_Vib_ ENCoM: −0.287 kcal.mol^−1^. K^−1^), as visualized in Figure 4B. According to the MutationTester, the variant is likely disease-causing and potentially capable of affecting the function of the APP protein. Due to multiple line computational evidence support, which (according to ACMG/AMP criteria) should be considered supporting evidence of pathogenicity (PP3) (Schwarz et al., 2014).

Given the variant’s proximity to known epilepsy-associated APP variants, and the patient’s seizure history, this case may contribute to the emerging genotype–phenotype correlation (Rujeedawa et al. 2021). Therefore, our analysis considers epilepsy as a comorbidity in AD, encompassing both sporadic and genetically determined forms. The underlying molecular mechanisms remain unclear, although the hyperexcitability potential of Aβ plaques is thought to play a key role (Cozza et al., 2023). According to current literature, seizures may be linked to common non-cognitive symptoms of AD, and evidence suggests that early onset of the disease is associated with earlier manifestation of epilepsy (a more severe clinical course) and accelerated cognitive decline (Cozza et al., 2023; Tang et al., 2016; Zheng and Koo, 2011). Data from a published cohort of patients (*n* = 171) with APP variants and concordant epilepsy indicate a prevalence of epilepsy of 20.3% in this group. Notably, at least one APP variant within the variant’s hotspot, p.Ala692Gly, has been reported to be associated with epilepsy (Cozza et al., 2023).

### 3.3 Limitations

Dosing, frequency, and duration of individual medications are incompletely documented. Given the extent of medication changes by multiple outpatient providers (over 10 years), the available records do not permit reconstruction of a comprehensive treatment history or the rationale for individual modifications.

Another notable limitation is the absence of early imaging, due to a 10-year archiving limit according to an implementing decree of the Ministry of Health, issued under the Act on Health Services. Accordingly, this case report is based on image descriptions from archived documentation, since the original scans are no longer retrievable.

Retrospective correlation of imaging studies with the established pathology was not feasible, which represents a main limitation of the present report.

## 4 Conclusion

According to the predictions, the extent of advanced amyloidosis suggests that our case may represent a gain-of-function (GoF) mutation, affecting the amyloidogenic pathway of APP. The presence of tauopathy was likely secondary, occurring in conjunction with intermediate Aβ pathology. This variant appears consistent with the patient’s phenotype – the concurrence of an APP gene variant with sporadic AD and atypical CAA is extremely unlikely. Due to fulfilling two supporting and two moderate ACMG/AMP classification criteria, the mutation should be considered likely pathogenic (LP) The presence of epilepsy in this case should be interpreted in the context of early-onset AD, independent of the underlying genetic etiology. Nevertheless, more evidence is needed to clearly demonstrate the pathogenicity (P) of the variant and its correlation with the exact clinical phenotype, in accordance with the stringent ACMG/AMP classification criteria (Douglas et al., 2014).

Our observation shows the clinical relevance of postmortem neuropathological verifications of genetic variants, even when they are predicted to be “benign” or VUS, and regardless of family history. Reported familial patterns may be misleading, especially in cases with very long clinical durations or the variant may have arisen *de novo*. The precise characterization of the variant can be highly relevant for the proband’s offspring and warrants appropriate clinical and genetic counselling.

## Data Availability

The original contributions presented in the study are included in the article/supplementary material, further queries can be directed to the corresponding author.

## Footnotes

1 See also www.alzforum.org/mutations/app.
